# Sedentary Work Postures and Mental Health Disorders

**DOI:** 10.1192/j.eurpsy.2026.11682

**Published:** 2026-07-31

**Authors:** N. Kotti, F. Dhouib, A. Kchaou, N. Kammoun, A. Mezghanni, M. Hajjaji, K. J. Hammami

**Affiliations:** 1 Occupational Health, Hedi Chaker University Hospital, Sfax; 2 Occupational Health and Safety Institute, Tunis; 3 Family Medicine, Faculty of Medicine, Sfax, Tunisia

## Abstract

**Introduction:**

Sedentary lifestyles at work, prevalent in administrative jobs, are associated with numerous health problems, such as mental health issues. However, this risk factor is often overlooked in occupational health policies.

**Objectives:**

The objectives of this study were to assess the level of sedentary behavior at work and analyze its impact on workers’ mental health.

**Methods:**

This is a cross-sectional, descriptive, and analytical study conducted over one year, from October 1, 2023, to September 30, 2024, in four faculties. Physical inactivity was assessed using the OSPAQ questionnaire, and mental health using the HAD scale

**Results:**

A total of 96 administrative staff were included in our study, the majority of whom were women (65.6%) with an average age of 49.4 years. Among the subjects, 75% had at least one medical history, dominated by hypertension (51.04%). The average length of service was 21 years. Administrative staff spent an average of 5.18±1.31 hours/day in a sedentary position. Univariate analysis showed a significant association between the duration of sitting at work and the occurrence of certain anxiety (p < 0.001) and a higher depression score (r = 0.202; p = 0.049).

**Conclusions:**

This study highlights the worrying impact of sedentary postures at work on workers’ mental health, emphasizing the urgent need to integrate the fight against sedentary lifestyles into prevention strategies in the workplace.

**Disclosure of Interest:**

None Declared

